# Interest in using patient portals among adolescents in mental health care - a cross-sectional study

**DOI:** 10.1186/s12913-023-09823-8

**Published:** 2023-08-09

**Authors:** Martine Stecher Hornum, Aslak Steinsbekk, Torunn Hatlen Nøst

**Affiliations:** 1 Department of Mental Health, Faculty of Medicine and Health Sciences, Norwegian University of Science and Technology, Trondheim, 7491 Norway; 2 Department of Public Health and Nursing, Faculty of Medicine and Health Sciences, Norwegian University of Science and Technology, Trondheim, Norway; 3 Norwegian Centre for E-health Research, Tromsø, Norway; 4 Norwegian Advisory Unit on Complex Symptom Disorders, St. Olavs hospital, Trondheim University Hospital, Trondheim, Norway

**Keywords:** Patient portals, eHealth, Patient activation, eHealth literacy, Electronic health records, Adolescent mental health care

## Abstract

**Introduction:**

Adolescents in mental health care may benefit from using patient portals to access personalised information about their health and treatment. While no studies have considered the interest in using patient portals among adolescents in mental health care, factors such as patient activation, self-reported health, depressive symptoms, diagnosis, healthcare utilisation, and eHealth literacy have been found to be associated with interest in and use of patient portals in other patient groups. Therefore, the aim was to explore the associations between interest in using patient portals and patient activation, self-reported health, depressive symptoms, diagnosis, healthcare utilisation and eHealth literacy among adolescents in specialist mental health care.

**Methods:**

A cross-sectional study among adolescents between 12 and 18 years of age receiving or having received treatment at four different specialist child and adolescent mental healthcare services across Norway. The adolescents´ answers to the questionnaire were linked to data on their healthcare utilisation and ICD-10 diagnoses from the Norwegian Patient Registry. The data were analysed using descriptive statistics and bivariate tests.

**Results:**

The 53 adolescents who participated, had a mean age of 15 years and 68% of them identified as female. Two out of three (64%) were interested in using patient portals. Most of the factors were not associated with interest in using patient portals. However, adolescents with mental and behavioural disorders (F diagnoses, 75% interested) were more interested in using patient portals compared to those with symptoms and signs involving cognition, perception, emotional state, and behaviour (R diagnoses, 31% interested).

**Conclusion:**

Except for mental health diagnosis, this study did not identify any specific factors likely to impact patient portal interest among adolescents in specialist mental health care.

## Background

Adolescents with mental health problems have access to a number of open and general websites targeting their situation [1]. While such websites are valuable, they do not provide information specifically about the adolescents’ own health situation and treatment as registered by healthcare services. One way to get access to such personalised information is through patient portals [2]. Patient portals, accessible via an app or website, allow patients to access features such as viewing their electronic health records, scheduling appointments, renewing prescriptions, accessing patient-specific educational material, and sending secure messages to healthcare providers [2]. Previous studies report that adults in mental health care and adolescents outside mental health care, experience benefits from using patient portals including increased trust in their healthcare providers and greater engagement in their care [3–5]. Thus, there might be advantages of having adolescents in mental health care start to use patient portals, and knowledge of the factors that influence the interest in using patient portals among adolescents in mental health care is therefore needed.

Studies on adult patient groups have shown that patients who take a more active role in their care (i.e., have a high level of patient activation) are more likely to use patient portals [6, 7]. Similarly, a study reported that adults in mental health care experienced an improvement in their patient activation after being granted access to a patient portal [5]. Furthermore, adolescents in mental health care who used an app developed to improve patient activation reported satisfaction and motivation with its use [8].

Other factors associated with interest in and actual use of patient portals among adult patients are related to healthcare utilisation [2, 9, 10], overall health status [11], and the severity of depressive symptoms [9, 10]. Users of patient portals in the general adult population are described as having poorer health and greater healthcare utilisation than non-users [2]. Moreover, the severity of depressive symptoms has been linked to increased healthcare utilisation among adolescents in mental healthcare [12].

Knowledge of how to use digital health services, such as patient portals, often called eHealth literacy, is also considered a relevant factor for adults´ awareness and use of patient portals [13, 14]. Even though today´s adolescents have been referred to as “digital natives”, they do not necessarily have the competencies to evaluate online health information [15, 16]. Deficiencies in finding, evaluating, and applying online health information, i.e., a lower eHealth literacy, also influenced adults´ use of patient portals [17], and adolescents´ online health information-seeking behaviours [18].

While no previous studies have considered whether adolescents in mental health care are interested in using patient portals and the factors that may influence their interest [19], such insight is important for the design, implementation, and clinical use of patient portals for adolescents in mental health care. Based on the relevance of the factors identified in previous research described above, the aim of this study was to explore the association between interest in using patient portals and the level of patient activation, overall health status, depressive symptoms, eHealth literacy, diagnosis, and healthcare utilisation among adolescents in specialist mental health care.

## Methods

A cross-sectional study was conducted between April and September 2022. The STROBE guidelines for observational studies were consulted during the design and reporting of this cross-sectional study [20].

### Setting

The study was conducted in Norway, a Northern European country with universal health coverage providing both specialist and primary health care including mental healthcare services [21]. Almost 65,000 children and adolescents receive specialist mental health care yearly in Norway, with outpatient care being the dominant treatment option [22]. The latest numbers from 2021 show that most adolescents receiving specialist mental health care in Norway either had a mental or behavioural disorder, chapter F in the ICD-10 coding (57%) or had symptoms and signs involving cognition, perception, emotional state and behaviour, part of chapter R in the ICD-10 coding, (40%) [22, 23].

In Norway, all citizens can access the national digital patient portal (www.helsenorge.no), and some healthcare services have their own digital patient portal solutions in addition. Patient portals offer a range of features, including access to information from one’s electronic health record such as test results and medical notes, prescriptions refill requests, appointment booking, asynchronous patient-provider communication, and online consultations.

Until the age of 12, parents normally have access to most information shared in their child´s patient portal. From the age of 12, adolescents get more rights in determining the information they will share with their parents [24, 25]. From the age of 16, which is the age of majority under health regulations in Norway, adolescents can access all their information in their patient portal [26, 27].

### Recruitment and participants

The eligibility criteria were adolescents aged 12 years or older currently receiving or had received treatment in the last three months at one of four different specialist child and adolescent mental healthcare clinics in Norway. The clinics were purposefully chosen to cover the various parts of Norway.

Eligible participants were identified by administrative staff at the clinics, who also wrote the patients’ names and addresses on pre-packed envelopes. The envelopes contained an information letter and consent form, the questionnaire, and a prepaid return envelope. After three weeks, a reminder was sent to all participants, asking them to disregard the request if they had already responded.

### Data collection

For participating adolescents aged 12 to 15, consent from both parents was required for their child´s participation. Consequently, for adolescents between ages 12 and 15, the questionnaire had to be answered on paper and returned by mail as no electronic solution existed that allowed for the practice of double parental consent. Adolescents aged 16 years or older, i.e., above the age of majority according to health regulations in Norway, were able to consent themselves. Therefore, adolescents aged 16 years or older could choose to either answer the questionnaire on paper or scan a QR code and answer the questionnaire digitally.

The questionnaire used in this study primarily included validated scales and questions from previously published studies on patient portal use. It was piloted on three adolescents aged 12–17, two of whom had prior experience with mental health care. Feedback from the participants of the pilot was used to make refinements and ensure clarity. The questionnaire began with a definition of a patient portal: *“A patient portal is an app or website that provides digital access to healthcare services. It may give you access to information from your health record that your healthcare providers have shared with you, such as test results and visit summaries. It may also provide features like appointment scheduling and the ability to contact healthcare providers for ongoing treatment. An example of a patient portal that is accessible to all residents in Norway is*www.helsenorge.no. *Additionally, many general practitioners also use various types of patient portals.”*

The adolescents aged 16 years or above, or their parents for those younger than 16 years, consented to the linkage of their questionnaire answers to data from the Norwegian Patient Registry (NPR), which included information on their contacts with specialist mental health care and their registered ICD-10 diagnoses.

### Measures

The question measuring interest in using patient portals was based on a Belgium study [28], and read as follows: *“Please indicate how strongly you agree or disagree with the following statements: I am interested in using a patient portal”*. Participants could select one of the following response options: “Strongly agree”, “Agree”, “Disagree” or “Strongly disagree”. The responses were then recoded into a binary variable, categorising “Interested” for answers “Strongly agree” or “Agree” or “Not interested” for “Disagree” or “Strongly disagree”.

Patient activation was assessed using the validated Norwegian version of the Patient Activation Measure (PAM®-13) [29]. It comprises 13 statements to which participants indicate their level of agreement on a four-point Likert scale, transformed to a score ranging from 0 to 100. The PAM®-13 has been employed in studies on eHealth interventions [30], patient portal use [31], and patient populations from the age of 12 [32]. The scores were categorised into four levels: level 1 (score 0–47), level 2 (score 47,1–55,1), level 3 (score 55,2–72,4) and level 4 (score 72,5-100) [33, 34].

Self-reported overall health was assessed using a single question: *“In general, how would you rate your health?”* with the following five response categories: “Excellent”, “Very good”, “Good”, “Fair” and ”Poor” [35, 36]. Self-reported health has previously been used to evaluate general health in various studies of adolescents, including adolescents in the Nordic countries [37, 38].

Depressive symptoms were assessed using the Patient Health Questionnaire-8 (PHQ-8), which is a validated adaptation of the original PHQ-9 developed for diagnosing and assessing depression. The PHQ-8 has also been used on adolescents and in Norwegian studies [39–43]. The PHQ-8 consists of eight items, and participants were asked to indicate the extent to which certain symptoms had bothered them for the last two weeks using the following response options: “not at all”, “several days”, “more than half the days” and “nearly every day”. The total score of the answers ranged from 0 to 24 and was categorised into four levels: minimal or mild depressive symptoms (0–9), moderate depressive symptoms [10–14], moderately severe depressive symptoms [15–19] and severe depressive symptoms [20–24].

The questionnaire also included one domain from the eHealth Literacy Questionnaire (eHLQ), a questionnaire consisting of seven domains, that has been validated in Danish and previously used in a Norwegian context [44–46]. The domain “Using technology to process health information” included five questions and was used as a measure of eHealth literacy. Participants were asked to indicate their level of agreement on a 4-point ordinal scale ranging from “strongly agree” to “strongly disagree”. The average score was calculated and then categorised into quartiles, as there is no universally established used cut-off for this domain.

Specialist mental health care contacts were counted as either: below 10, between 10 and 30, or above 30 contacts, within the last 12 months. This includes all activities and encounters where the patient was present, such as consultations, online consultations, and telephone contact between the provider and the patient. However, the code for indirect patient contact was excluded. The ICD-10 diagnoses were grouped into the two chapters most commonly used in Norwegian specialist mental health care for adolescents: “Mental and behavioural disorders” (Chapter F) or “Symptoms, signs and abnormal clinical and laboratory findings, not elsewhere classified” (Chapter R) [22]. Adolescents with more than one diagnosis were categorised according to their primary diagnosis.

Socio-demographic information was self-reported and included age, gender with three categories (female, male and other), the participants’ school or daytime activity, and parents’ highest education.

### Sample size

This study was part of a larger quasi-experimental study that aimed to assess the difference in PAM-13 scores between users and non-users of a comprehensive patient portal. The power calculation for the larger study showed that 588 adolescents were needed to observe a difference of at least 4 points between groups, with a standard deviation of 15, an alpha of 0.05, a power of 80%, and an allocation ratio of 1:3. We managed to obtain information on 623 adolescents, and they were invited to account for non-responses.

### Analysis

Data from the questionnaire were linked to data from the Norwegian Patient Registry (NPR) on participants´ contacts with specialist mental health care and ICD-10 diagnoses. Descriptive statistics were performed to present the characteristics of the participants, and bivariate analysis using chi-square tests was performed to examine the associations between the adolescents’ interest in using a patient portal and their levels of patient activation, self-reported overall health, depressive symptoms, eHealth literacy (domain 1), ICD-10 diagnoses, and the use of specialist mental health care. All analyses were conducted in STATA 17 (www.stata.com).

## Results

In total, 623 adolescents in adolescent mental health care were sent an invitation to participate in the study, and 53 (8.5%) of them responded positively. 70% of the adolescents answered the questionnaire themselves, 28% did so with a parent, and 2% with another person. The majority of the adolescents had mothers (74%) and fathers (68%) with higher education (defined as education after high school).

The mean age of the adolescents was 15 years (SD 1,5), and 68% of them identified as female (Table 1).

**Table 1:** Patient characteristics and the proportion reporting interest in using a patient portal for each characteristic. N (%) or mean (SD)

Characteristics of the adolescent	N (%)	Interested in using patient portals (%)
Total number of adolescents	53 (100%)	64%
Age	53 (100%)	
- 12–13 years	7 (13%)	57%
- 14–15 years	18 (34%)	67%
- 16 years	16 (30%)	63%
- 17–18 years	12 (23%)	67%
Self-reported gender	53 (100%)	
- Female	36 (68%)	61%
- Male	7 (13%)	71%
- Other	10 (19%)	70%
School or daytime activity	53 (100%)	
- Elementary school	4 (8%)	50%
- Junior high school	22 (42%)	64%
- High school	24 (45%)	63%
- Other (e.g., Offer from the follow-up service or working)	3 (5%)	100%

Sixty-four percent (34 of 53) of the adolescents expressed interest in using a patient portal (Table 1). Those between the ages of 12–13, as well as those in elementary school, reported the lowest interest.

No association was found between interest in using patient portals and the adolescents´ levels of patient activation (PAM-13), depressive symptoms (PHQ-8), eHealth literacy (eHLQ-1), or self-reported health (Table 2).

**Table 2:** Association between interest in using patient portal and patient activation (PAM-13), depressive symptoms (PHQ-8), use of technology to process health information (eHLQ-1) and self-reported health

	N	Interested in using patient portals. N (%)	P-value
Patient Activation (PAM-13^a^)	53		0,964
- Level 1 (0–47)	10	6 (60%)	
- Level 2 (47,1–55,1)	8	5 (63%)	
- Level 3 (55,2–72,4)	22	15 (68%)	
- Level 4 (72,5-100)	13	8 (62%)	
eHealth literacy (eHLQ-1^b^)	53		0,795
- Lower quintile	26	16 (62%)	
- Middle quintile	10	6 (60%)	
- Upper quintile	17	12 (71%)	
Depressive symptoms (PHQ-8^c^)	52		0,990
- Minimal and mild depressive symptoms (0–4)	15	10 (67%)	
- Moderate depressive symptoms (10–14)	15	10 (67%)	
- Moderately severe depressive symptoms (15–19)	13	8 (62%)	
- Severe depressive symptoms (20–24)	9	6 (67%)	
Self-reported health	52		0,720
- Excellent and very good	5	4 (80%)	
- Good	15	10 (67%)	
- Fair	24	14 (58%)	
- Poor	8	6 (75%)	

^a^ = PAM13 is a scale ranging from 0 to 100, with 100 indicating the highest patient activation which is commonly divided into four levels

^b^ = eHLQ-1 is a domain in the eHLQ-scale and consists of five items scored on a range from 1 to 4. The average score was calculated and categorised into quartiles as no commonly used cut-off exists

^c^ = PHQ-8 is a scale ranging from 0 to 24 with categories based on the frequency and severity of depression symptoms

More adolescents with mental and behavioural disorders (F-diagnoses, 75%) were interested in using patient portals compared to those with symptoms and signs involving cognition, perception, emotional state, and behaviour (R-diagnoses, 31%) (Table 3). There was no association between the frequency of contacts with specialist mental health care within the past 12 months and the level of interest in using patient portals. However, adolescents who had fewer visits tended to be less interested.

**Table 3:** Association between interest in using a patient portal and registry data on the type of principal ICD-10 diagnosis and contacts with specialist mental health care

	N	Interested in using patient portals. N (%)	P-value
Principal diagnosis (ICD-10 codes)			0,004
- R-diagnosis	13	4 (31%)	
- F-diagnosis	40	30 (75%)	
Contacts with specialist mental healthcare last 12 months			0,153
- < 10	14	6 (43%)	
- 10–30	25	18 (72%)	
- > 30	14	10 (71%)	

## Discussion

Among the participants in this study, two out of three were interested in using patient portals, with minor variations depending on adolescent characteristics. The only factor associated with interest in using patient portals was their diagnosis, where adolescents with a mental or behavioural disorder (F-diagnoses) were more than twice as interested compared to those with symptoms and signs involving cognition, perception, emotional state, and behaviour (R-diagnoses).

Due to the low response, this study cannot be used to say anything about the proportion of adolescents in specialist mental health care who are interested in using patient portals. Studies conducted in other populations have reported that interest in or usage of patient portals ranged from between 40 and 90 % [28, 47, 48].

The primary conclusion of this study was that interest in using patient portals was not associated with the factors: patient activation, eHealth literacy, depressive symptoms, self-reported health, or the number of contacts with specialist mental health care. This finding contradicts earlier research that demonstrated associations between patient portal interest or usage in other populations, as presented in the introduction. Although this study cannot provide definitive explanations as to why this is the case, some suggestions can be offered. Adolescents´ limited access to digital healthcare services and personal health information [49, 50], may make them more generally interested in using patient portals regardless of their health status or personal characteristics. Additionally, since adolescence is a period of profound cognitive, emotional, and physical changes, it is possible that other unmeasured factors may be more important for adolescents’ interest in patient portals.

The type of diagnosis was the only factor found to be associated with interest in using patient portals. No other comparable study has been found, but a study of adult military veterans in mental health care [51] who found differences in the interest in using patient portals depending on the diagnosis. For example, individuals with depression, anxiety, and military sexual trauma were more interested in using patient portals compared to those diagnosed with e.g., bipolar disorder and substance use. Adolescents with an R diagnosis typically receive this diagnosis at the beginning of their care trajectory or when a specific mental health diagnosis is not found [52]. As those with an R-diagnosis showed less interest, it suggests that patient portals may be more relevant for those who have been in treatment for a longer period and have been diagnosed with a mental or behavioural disorder. This corresponds with previous studies reporting that patients with a chronic disease [14] and comorbidity [2] were more likely to use patient portals than those without. However, the finding that the number of contacts was not clearly associated with interest in patient portal use seems to contradict this. Yet, upon closer examination of the number of contacts, it becomes evident that those with less than 10 contacts are less interested in using patient portals. A post-hoc analysis comparing those with less than 10 contacts (43% interested) to those with 10 or more contacts (72% interested) was conducted, revealing a difference of 29 % points (95% confidence interval − 0,6 to 59, P = 0,053).

### Strengths and limitations

To the best of our knowledge, this study is the first to investigate factors associated with interest in patient portal use among adolescents in mental health care that have been shown to be relevant in other patient populations.

To provide insight into the factors affecting the adolescents´ interest in using patient portals, self-reported measurements were found to be suitable. To compensate for potential limitations, validated questionnaires used in previous research were used.

One noteworthy limitation is the low response rate. However, recruiting participants is a common challenge in this patient population [53]. While using alternative recruitment methods other than having the administrative staff at the clinics identify respondents could potentially have resulted in more responses, we were not allowed to directly recruit the adolescents ourselves. Moreover, another possible explanation for the low response rate may be the requirement to obtain two parental consents, as well as the fact that the questionnaires were sent by post.

## Conclusions

In this first explorative study investigating factors associated with the interest of adolescents in specialist mental health care in using patient portals, it was found that, except for mental health diagnosis, none of the other included factors were associated with interest in patient portal use. Larger studies are required to confirm this finding.

## Data Availability

The data that supports the findings of this study are not openly available due to the regulation of the Regional Ethical Committee, that we must secure the anonymity of the informants, but an anonymised data file is available from the corresponding author upon reasonable request. The data can be found at the Department of Mental Health at the Norwegian University of Science and Technology in Trondheim, Norway.

## Abbreviations

eHLQ: eHealth literacy Questionnaire
ICD-10: International Classification of Diseases 10th Revision
NPR: Norwegian Patient Registry
PAM-13: Patient Activation Measurement – 13 items
PHQ-9: Patient Health Questionnaire – 9 items
